# Development and Validation of the “Basic Oral Health Assessment Tool” (BOHAT) for Nondental Health Care Professionals to Use With the Indian Adult Population: Protocol for a Mixed Methods Study

**DOI:** 10.2196/63480

**Published:** 2025-02-13

**Authors:** Amitha Basheer N, Praveen Jodalli, Shishir Shetty, Ramya Shenoy, Ashwini Rao, Mithun Pai, Inderjit Murugendrappa Gowdar, Sultan Abdulrahman Almalki

**Affiliations:** 1 Department of Public Health Dentistry Manipal College of Dental Sciences Mangalore Manipal Academy of Higher Education Manipal India; 2 Department of Oral and Craniofacial Health Sciences College of Dental Medicine University of Sharjah Sharjah United Arab Emirates; 3 Department of Preventive Dental Sciences College of Dentistry Prince Sattam Bin Abdulaziz University Al Kharj Saudi Arabia

**Keywords:** oral health assessment tool, oral health, screening, nondental health care professionals, primary health centers, India, tool validation, health care training, mixed methods research

## Abstract

**Background:**

Oral health is a significant indicator of general health, well-being, and quality of life. The prevention of oral health problems requires periodic inspection of the oral cavity. Routine oral health examinations at the individual level appears to be one way to deliver quality oral health care but are too often missed as an opportunity for improved oral health in the nondental health care setting in India. This is because of limited training and inaccessible or lack of specialized oral health assessment tools.

**Objective:**

This study will focus on the development, validation, and implementation of the Basic Oral Health Assessment Tool (BOHAT) to improve the oral health assessment capabilities of nondental health care professionals and thus contribute to improved overall health outcomes of the Indian adult population.

**Methods:**

This study will be a mixed methods, multistage study conducted in 3 stages. The study will be conducted with 708 nondental health care professionals in 33 Primary Health Centers (PHCs) of Mangalore Taluk, Karnataka. Ethical approval was sought from the institutional ethics committee of Manipal College of Dental Sciences Mangalore. Informed consent will be obtained from every participant prior to the study. A literature review and qualitative interviews will be used for item and domain generation with respect to BOHAT, and an expert panel review and pilot testing will be used to refine the items and domains. Finally, statistical analyses will be conducted to validate the reliability and consistency. The second phase will involve capacity building and user experience exploration through comprehensive training for nondental health professionals using audio and visual aids, with hands-on learning methodologies including relevant feedback processes in the form of focus group discussions. The third stage will check the effectiveness of BOHAT regarding the changes in knowledge, attitudes, and practices through pre- and posttraining questionnaires, which will then be followed by a retention analysis 3 months later.

**Results:**

As of January 20, 2025, the study is in its preliminary phase: “Substage A: Item and Domain Development.” We have received institutional ethics committee and Institutional Protocol Approval Committee approval for the study. Data collection procedures have not started yet. The study is progressing as per the planned timeline.

**Conclusions:**

The BOHAT study holds considerable potential to promote oral health care through collaborative and interdisciplinary approaches. It will facilitate early diagnosis, timely referrals, and comprehensive care by integrating assessment actions for oral health into routine practices of nondental primary health care professionals.

**International Registered Report Identifier (IRRID):**

PRR1-10.2196/63480

## Introduction

Oral health is a significant indicator of general health, well-being, and quality of life [1]. The functioning of the oral cavity, teeth, and orofacial structures, which enables people to perform fundamental bodily activities like eating, respiration, and speaking, is commonly referred to as oral health, and it undergoes changes throughout the various stages of life, from early childhood to advanced age [2,3]. Oral health problems can have a significant effect on people’s quality of life, interpersonal connections, general well-being, and self-esteem [4]. Therefore, emphasizing the importance of preventive measures and early detection of oral diseases becomes crucial in mitigating the likelihood of developing subsequent challenges impacting both oral and overall health.

Oral health assessments serve as a good indicator of disease risk, the proper management of existing disease, and even the improvement of health outcomes because of appropriate oral health care [5]. Prevention of oral health problems requires periodic inspection of the oral cavity. Routine oral health examinations at the individual level appear to be one way to deliver quality oral health care but are too often missed as an opportunity for improved oral health in the nondental health care setting [6]. Health care professionals, from the allopathic doctor (physician and surgeon) to Ayurveda, yoga, naturopathy, Unani, Siddha, and homeopathy (AYUSH) practitioners; nurses; auxiliary nurse midwives; and community health workers (CHWs) [7], are very essential for the delivery of holistic health care but have limited capacity to comprehensively assess oral health issues and subsequently intervene because of significantly limited training on its own as well as inaccessible or lack of specialized supportive tools adapted to suit the population. This might ultimately delay the diagnosis and institution of appropriate interventions for oral health conditions in such patients and increase the possibility of further disease progression, which will then compromise overall health outcomes.

The separation between oral health and general health care practices may contribute to the insufficient integration of oral health assessments into routine health care protocols, limiting the overall effectiveness of health care delivery. Screening of oral health, early detection and triage of oral health issues, and immediate referral to dental specialists ultimately require the integration of frontline health care professionals [8]. Several instruments for health care providers to evaluate oral health have been developed over time. A few of these tools are the Holistic Reliable Oral Assessment Tool (THROAT) [6], Revised Oral Assessment Guide [9], Oral Health Assessment Tool (OHAT) [10], and Oral Assessment Guide [11]. However, the tools in current use lack reference pictures, challenging the completeness, relevance, and clarity of wording [12]. The Federation Dentaire Internationale and International Consortium for Health Outcomes Measurement collaboration developed a preliminary standard core set of oral health outcome measures for adults with a primary focus on periodontal disease and caries. The subsequent action is to conduct a validation and feasibility study in different clinics seeking to fine-tune the Adult Oral Health Standard Set more, and studies to do that are ongoing [13].

To bridge this gap, this research aims to develop, validate, and implement a Basic Oral Health Assessment Tool (BOHAT) that is tailored for use by nondental health professionals working with populations aged 18 years and older in India. BOHAT will be a screening tool for health professionals who are not dentists, to enable rapid identification of the need to refer patients for dental consultation. This tool will further empower nondental health care professionals to perform effective oral health assessments, thereby addressing an emerging critical need for holistic health care approaches at large. This study will focus on the development, validation, and implementation of BOHAT to improve the oral health assessment capabilities of nondental health care professionals and thus contribute to improved overall health outcomes of the Indian adult population.

## Methods

### Stages

**Figure 1:**
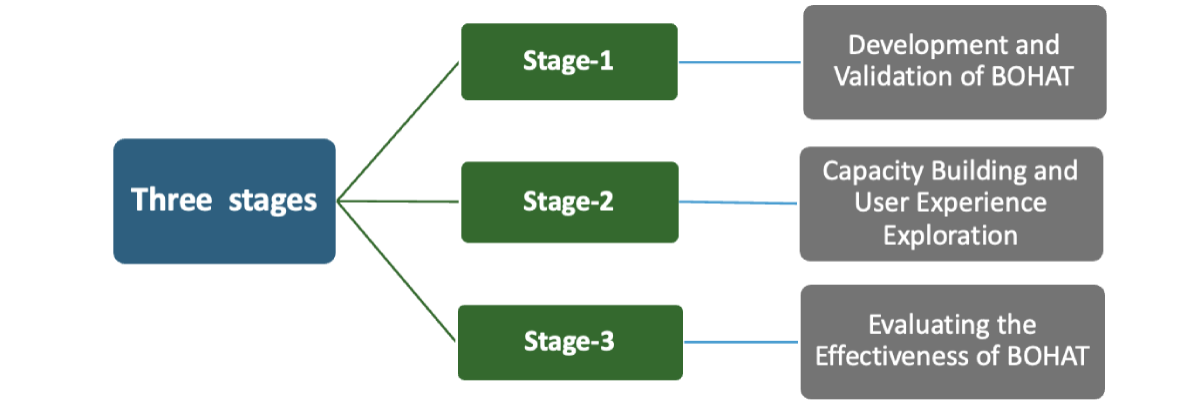
Multiple stages of the study. BOHAT: Basic Oral Health Assessment Tool.

This study will be a mixed methods, multistage study conducted in 3 stages. Stage 1 focuses on the development and validation of the tool. Stage 2 involves capacity building and user experience exploration, and stage 3 aims to check the effectiveness of BOHAT using a pre-post intervention study (Figure 1).

### Study Setting

The study will be carried out in Primary Health Centers (PHCs) located within Mangaluru Taluk of Dakshina Kannada District. This selected study setting will enable insights into the applicability and effectiveness of the developed tool, BOHAT, under Mangaluru Taluk’s health care infrastructure. In this approach, oral health assessment practices will be examined more precisely and contextually in relevance to the geographic area selected. PHCs in this region will be the primary sites for data generation, data collection, pilot testing, validation studies, and execution of training programs for nondental health professionals. Approval to conduct the studies in PHCs will be obtained from the District Health Officer, Dakshina Kannada, Karnataka.

### Sampling Technique and Sample Size

The sampling technique used in this study is census sampling or complete enumeration, which includes all 708 nondental health care professionals working in the 33 PHCs of Mangalore Taluk. This covers professionals from both urban (10 PHCs) and rural (23 PHCs) areas, ensuring that this target population is widely and appropriately represented.

Nondental health care professionals, including allopathic doctors (physicians and surgeons), AYUSH practitioners, nurses, auxiliary nurses, midwives, and CHWs [7]. Within the urban PHCs, there are 216 nondental health care and allied health care professionals, while the rural PHCs have a workforce of 488 nondental health care and allied health care professionals. The inclusive nature of this study aims to involve all 708 nondental health professionals across all 33 PHCs in Mangalore Taluk who provide their consent for participation.

### Study Framework

BOHAT will be designed as a paper-based instrument with the inclusion of reference pictures. These reference images serve as visual aids to enhance and facilitate the oral health assessment process**.**

### Stage 1: Development and Validation of BOHAT

**Figure 2:**
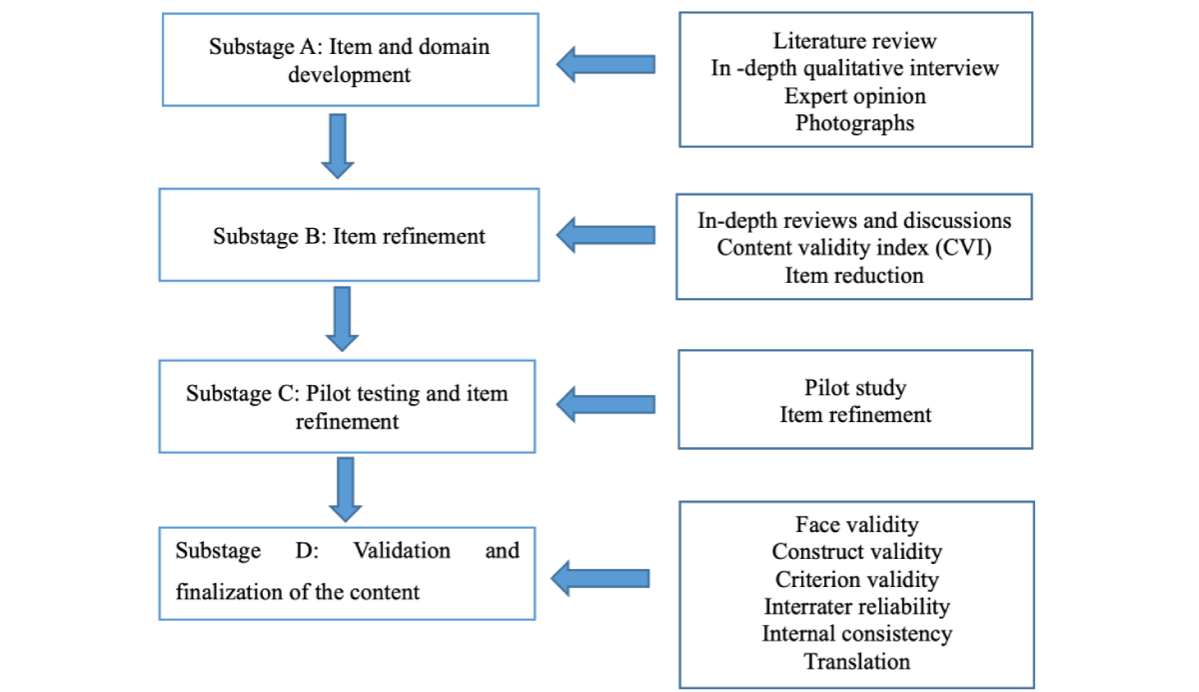
Conceptual framework for the development and validation of the Basic Oral Health Assessment Tool (BOHAT).

Stage 1 has 4 substages (Figure 2).

#### Substage A: Item and Domain Development

A literature search will be conducted to obtain data on oral health, oral hygiene, and common dental problems among Indian adult population. An in-depth qualitative interview will be undertaken with nondental health professionals to explore the challenges and complexities inherent in oral health assessment. Additionally, this approach aims to identify key domains that should be incorporated into the BOHAT. Collaboration with oral health care experts from public health dentistry, oral medicine and radiology, oral pathology, oral surgery, periodontics, endodontics, and prosthodontics will identify relevant and essential oral health problems specific to the Indian adult population.

Photographs for BOHAT will be sourced from patients availing of camps organized by the Department of Public Health Dentistry, Manipal College of Dental Sciences, Mangalore. These photographs will cover a vast spectrum of conditions and scenarios pertaining to oral health, thereby presenting a sample of what nondental health professionals might encounter during their assessments. Prior to photographing, proper ethical consent will be obtained from the patients. The photographs will be taken personally by the principal investigator after completing training in intraoral photography. This will help maintain uniformity so that image collection and image capture follow standardized procedures.

The photographic setup for the intraoral photos will include a Canon EOS 90D camera with a Canon EF 100mm f/2.8L Macro IS USM lens. Adequate lighting in the mouth will be provided using a Canon MR-14EX II Macro Ring Lite, which provides lighting without any harsh shadows and gives the best chance of even illumination. The images will have a resolution of 32.5 megapixels and will be saved in both RAW and JPEG formats. This ensures they will provide sufficient detail and can be modified to suit the intended use. The minimum and maximum ISO standards will be set between 100 and 200, aperture will be set at f/22 to ensure enough depth of field, and the shutter speed will be set between 1/125 and 1/200 seconds in order to minimize motion blurs. The custom white balance will be by intraoral light. Each photo will be captured from a distance between 30 cm and 40 cm, and since the ring flash will be used, a fixed distance will be sustained. Each file will be numbered, kept in encrypted digital storage, and secured to ensure data integrity.

Images will be identified and selected by subject matter experts according to the relevance, clarity, and appropriateness of content in relation to the goals of BOHAT. Each image will be critically evaluated to ensure that the wide spectrum of oral health conditions and scenarios that the nondental health care professionals may come across during assessment is captured. This includes ensuring there is sufficient detail and contextual setting in the images for both normal and pathological conditions. Images will further be categorized based on the diagnostic significance, such as specific oral issues like dental caries, periodontal diseases, or mucosa lesions. Selected images will then be further checked for quality assurance to ensure they are educationally valuable, appropriate for educational purposes, and meet ethical standards.

#### Substage B: Item Refinement and Item Reduction

The items on the BOHAT will undergo rigorous examinations, review, and engagement with the expert panel so that remarks on issues related to clarity, relevance, and appropriateness are captured. Items will also be subjected to systematic refining through discussions of the panels with respect to clarity, relevance, and appropriateness. To evaluate the test’s content validity, the content validity index (CVI) will be computed to measure the relevance of each item. Items with a low CVI (eg, item-level CVI<0.78) will be revised to improve their validity, or only the most relevant items will be retained. There will also be feedback from various stakeholders to enhance the contextual and functional appropriateness of the tool.

#### Substage C: Pilot Testing and Item Refinement

We will approach 20 nondental practitioners to pilot test the tool for its usability and clarity. This feedback will be used to provide input on the validity and reliability of the tool. Based upon the results of the pilot testing, these improvements will fill in the gaps before formal validation occurs. Descriptive statistics along with qualitative coding of each feedback will be used for initial evaluation of the tool’s performance.

#### Substage D: Measurement Properties and Finalization of the Content

##### Consensus-Based Standards for the Selection of Health Measurement Instruments Taxonomy

Development and validation will be based on the COSMIN (Consensus-Based Standards for the Selection of Health Measurement Instruments) taxonomy [14]. The COSMIN framework will ensure that this newly developed tool adheres to high methodological standards and permits a rigorous assessment of its primary measurement properties including validity, reliability, and responsiveness.

##### Validity Testing

For face validity, feedback will be obtained from dental professionals, nondental health professionals, and other stakeholders to establish whether the tool is easy to read, accurate, and relevant. A qualitative assessment will follow, to ensure that the tool is fit for purpose and feasible for end users.

**For content validity,** content experts will assess the CVI of each item for relevance, clarity, and representativeness. These experts will provide CVI scores as a basis for revision, ensuring objectivity through systematic thresholds, for instance, an item-level CVI≥0.78.

Construct validity of BOHAT will be thoroughly assessed to confirm the extent to which the measure assesses oral health assessment capacity for nondental health care professionals. Exploratory factor analysis will be conducted to determine the underlying structure of the tool by grouping items into the dimensions of knowledge, attitudes, and practices (KAP) of oral health assessments. The appropriateness of the factor structure will be considered with the help of statistical tests such as the Kaiser-Meyer-Olkin test as well as the Bartlett test of sphericity. Confirmatory factor analysis will then validate the identified structure. A root mean square error of approximation ≤0.08 and comparative fit index ≥0.90 will be used to assess fit.

To determine criterion validity, each BOHAT item will be assessed against standardized criteria derived from the World Health Organization (WHO) oral health survey [15]. Each item will be directly mapped to the corresponding items or domains in the WHO oral health survey. For instance, the items measuring dental caries, periodontal health, and oral hygiene status will be benchmarked against the WHO criteria on such conditions. BOHAT and the WHO oral health survey will be administered to the same sample of participants. The sensitivity and specificity parameters will be determined as a statistical measure or estimate of validity for each considered BOHAT item in comparison with the WHO criteria for oral health conditions. Sensitivity and specificity values will show the diagnostic accuracy of the items.

Positive and negative predictive values will provide measures representative of usable or functional utility for each variable under study. Reference variables that form a strong connection with either a positive or negative outcome might be derived from those that create the curves and an area under them in the receiver operating characteristic (ROC) curve using the area under the curve (AUC) as an overall measure of accuracy, such that AUC values ≥0.8 indicate good performance. Items with low sensitivity or specificity will either be reconsidered or removed to obtain the most diagnostically relevant and accurate items.

##### Reliability Testing

For internal consistency, the Cronbach α will be computed and used as a measure of internal consistency with respect to the degree to which something is being measured as intended. The Cronbach α ranges from 0 to 1. An α near 1 indicates a high internal consistency of the items. Standardization of the scoring criteria for BOHAT, on a consensus basis through collective opinion and expert opinion on subject matter issues, will allow uniformity and objectiveness in assessments.

For interrater reliability, different nondental health professionals will independently use BOHAT with the same patients, and the scores will be checked for their agreement. The ratings proposed by different raters may be statistically analyzed using metrics like the Cohen kappa or the intraclass correlation coefficient (high values [≥0.75] represent excellent agreement).

For test-retest reliability, BOHAT will be evaluated among the same participants at specific intervals to estimate score stability. This will be done using metrics reflecting continuity across time intervals such as intraclass correlations.

#### Responsiveness of BOHAT

Evaluating the responsiveness of BOHAT will be a vital element in determining the validity of the training for augmenting the oral health assessment skills of nondental health professionals. Differences in scores before and after the training will be assessed using the pre- and posttraining questionnaires as compared with the participants’ self-reported changes in confidence and competence of conducting oral health assessments. Focus group feedback will also be analyzed to obtain a sense of perceived skill improvements by participants. Distribution-based methods will be used to evaluate changes in pre- and postintervention scores, including calculation of the effect size (Cohen *d*) to measure the magnitude of change and the standardized response mean to evaluate BOHAT responsiveness to detect clinically meaningful changes. Statistical comparisons of pre- and posttraining scores will be conducted using paired *t* tests or Wilcoxon signed-rank tests. Additionally, minimal clinically important differences will be determined using ROC curve analysis to interpret thresholds of change in the BOHAT that are considered clinically significant.

#### Translation of BOHAT

Although BOHAT is primarily in the English language, compliance will ensure proper adaptation of BOHAT for application in Kannada-speaking regions. Adjustments could include providing explanations on linguistic and cultural settings whenever there is a need for clarification. The procedure will likely correspond to the systematic approach provided for the back-translation model by Brislin [16] and comprise the steps outlined in the following sections.

#### Forward Translation

The test items will be in English; however, key instructions, terms, and technical language will be translated into Kannada by a well-trained bilingual expert who is a native Kannada speaker. This step will provide nondental health care professionals who are comfortable with Kannada the capacity to understand the use and purpose of the tool with ease.

#### Review by an Expert Team

To ensure cross-cultural equivalence, all translated versions will be reviewed by a multidisciplinary team. A total of 3 oral health experts and nondental health care professionals who have experience with oral health assessments will check the cultural relevance of the terms and concepts with the research team. We will consult 5 nondental health care professionals for practicality and relevance of the tool in the Kannada-speaking health care setting. Any semantic and conceptual changes required will be undertaken based on their feedback, resulting in a first Kannada version of BOHAT.

#### Back-Translation

A second professional bilingual translator will then back-translate the preliminary Kannada version into English. This translator will be blinded to the original English version to avoid bias.

#### Comparison and Revisions

The back-translated English version of BOHAT will be compared with the original English version, during which contradictions or deviations will be detected. Revisions will be made when necessary to ensure that the Kannada clarifications correspond to the meaning of the original English version, hence preserving the integrity of the tool items.

#### Pilot Testing

A refined version of BOHAT in Kannada will be pilot tested with 20 nondental health practitioners in rural and urban PHCs. Each participant will pilot test the tool and provide feedback on items that appear to be unclear or culturally insensitive. The feedback for clarity and utility of additional Kannada content will help determine whether they can understand and use BOHAT properly. The participants will rate each item on a scale from 1 to 5 for relevance, clarity, and specificity. The feedback from this phase will guide further adaptation toward achieving semantic and content equivalence.

The English version will be the final version of BOHAT, supplemented by additional explanatory material in Kannada for users needing language support. This will ensure that the original tool stays in the intended format in English while remaining culturally relevant and accessible to Kannada-speaking health care professionals in the setting.

### Stage 2: Capacity Building and User Experience Exploration

#### Process

The training modules will have details on the purpose of BOHAT, administration procedure, and interpretation of the results. Experts will be given this module, and for content and face validity, they will be invited to classify the need for each question according to a 3-point Likert scale: “necessary,” “useful but unnecessary,” and “unnecessary.” Depending on the experts’ opinion, the content will be modified. The BOHAT training module will adopt a multimodal mode of learning: audio, visual, and audiovisual modes. This will ensure maximum benefits from training sessions so that better understanding and actual application in oral health assessment practices can be achieved.

The module for the training will include how to navigate the tool, interpret the results, and communicate the findings efficiently to patients. Hands-on training sessions will be conducted regarding using the tool. A focus group discussion will be conducted with nondental health professionals to explore the experiences of the participants, challenges faced by them, and perceptions about BOHAT’s usability.

#### Training Module

This training module is 4 hours long and will be delivered using a combination of audio, visual, and audiovisual aids. The comprehensive training on common problems of oral health among the adult population in India shall draw upon a variety of subjects to provide the necessary background to a health professional. The module will start with an overview of the role of health professionals in disseminating awareness regarding oral health problems and the importance of dental health in relation to overall health. The epidemiology of oral health in India will be presented with data on the most common oral health problems among adults.

The module will consider specific oral health problems often seen in adults. It will cover in-depth the processes of dental caries, periodontal diseases, and tooth sensitivity; the association between tobacco and oral health; potentially malignant disorders and oral cancer; other oral lesions; and the risk factors and lifestyle factors, like diet and lifestyle, affecting oral health. At the same time, emphasis will be placed on early detection and prevention. The module will cover potential solutions to improve access and further incentivize health professionals to incorporate oral health discussions into their practice. The training will be completed with key takeaways underscoring the importance of oral health awareness and preventive measures. An interactive question-and-answer session will be conducted, and participants will have time to share their experiences and discuss special concerns.

### Stage 3: Evaluating the Effectiveness of BOHAT

A validated questionnaire will be used by each nondental health professional before the training session to gauge their baseline KAP regarding oral health assessments. The same questionnaire administered after training will help assess the impact and effectiveness of BOHAT on the KAP of nondental health professionals. The scores obtained from the posttest of BOHAT will be compared with benchmarks as observed by dentists. This comparative assessment aims to establish the effectiveness of BOHAT through the tool’s degree of conformance with existing standards in dental practice. Retention analysis 3 months after the posttest will evaluate and measure the retained knowledge and acquired skills from the training sessions. Results of the retention analysis will rate how effective the training was when applied and in the long run to help modify or reinforce the practice of oral health assessment.

### Statistical Analysis

Chi-square tests will be used to compare the number and percentage of oral health problems detected in a timely manner by nondental health care professionals before and after the training. Referral frequencies before and after training will be used to measure the changes in early referral behavior. Paired *t* tests or Wilcoxon signed-rank tests will be used to analyze any differences found. We will create a reflected quantitative summary of baseline knowledge, and improvement in the KAP of oral health will be evaluated using validated pre- and posttraining questionnaires. Thematic analysis of the qualitative data obtained through the semistructured interviews and focus group discussions will help provide insights into improvements in collaboration with dental and health professionals.

Data from the training assessment and improvements in competence will be analyzed using paired *t* tests, and the reliability of BOHAT will be assessed using the Cronbach α. Referral logs will subsequently be analyzed to gauge increased accessibility to dental care, and trends over time will be evaluated using longitudinal data analysis methods such as linear mixed-effects models. Finally, retention analysis will be conducted 3 months after the training to examine the sustainability of acquired knowledge and skills measured using KAP questionnaires, and time effects will be analyzed using repeated measures ANOVAs.

### Ethical Considerations

The Declaration of Helsinki principles will be followed while conducting the research [17]. Ethical approval was sought from the institutional ethics committee of Manipal College of Dental Sciences Mangalore, Manipal Academy of Higher Education (reference number: 24023). Additionally, the Institutional Protocol Approval Committee of the Manipal Academy of Higher Education provided approval for the study (2300400101). Informed consent will need to be provided by every participant after verbal and written information regarding the study is given to them.

All participant data will be anonymized: Names, addresses, or any other forms of unique characteristic directly identifiable in any data collected will be removed from the data set prior to its analysis. In preparation for data coding, all participants will be allocated an ID code that links to personal details using a secure key held separately. For sensitive data that cannot be anonymized, such as qualitative responses or images, access to identifiable data will be strictly limited to authorized research team members. Electronic data shall be stored on secured, encrypted, password-protected devices. Physical records shall be kept in locked cabinets. There shall be no sharing of identifiable data beyond the research team, and the data shall be used only for research.

Participants will not receive monetary compensation for this study, but they will be compensated for any travel costs associated with their involvement in the study. No identifiable characteristics of the participants will be described in the manuscript, supplemental materials, or images.

## Results

### Study Status

As of January 20, 2025, the study was in the preliminary phase (Substage A: Item and Domain Development). The study will be carried out for a period of 42 months. We have received institutional ethics committee and Institutional Protocol Approval Committee approval for the study. The research team had a meeting with the District Health Officer, Dakshina Kannada District on January 10, 2025, and the study protocol was submitted to the District Health Officer for review. Data collection procedures had not started yet. The study was progressing as per the planned timeline.

### Dissemination

This study will be complemented by in-depth qualitative and quantitative analysis. The results of the study will then be presented at relevant scientific conferences and professional meetings as oral presentations and published in academic journals.

### Expected Outcomes

The expected outcomes of the study include timely identification of oral health problems and an increase in early referral behavior. This will improve oral health assessments by nondental primary health care providers and oral health KAP of these nondental primary health care providers, leading to enhanced collaboration with dental and other health professionals and improved training and competency. Increased access to dental care is also expected.

## Discussion

### Overview

Oral health problems can have a significant effect on people’s quality of life, interpersonal connections, general well-being, and self-esteem [4]. It is therefore important to address the role of preventive techniques and early diagnosis of oral pathologies in reducing the risk of developing further oral-health-related complications affecting not just oral but also general health. Therefore, oral health assessments are an important part of estimating disease risk, monitoring existing conditions, and improving oral health [5].

The current educational frameworks for these practitioners do not sufficiently cover oral health topics, leading to gaps in knowledge and practice [18]. Health care providers face problems effectively assessing and addressing oral health issues in the population because of a lack of adequate training and inaccessibility of tools specialized for the Indian population.

The Brief Oral Health Status Examination allows carers to monitor the dental health of patients in aged care institutions who are cognitively challenged as well as those who are not. However, the study was conducted in only one nursing home, and the sample size was small [19]. THROAT is another instrument for the oral evaluation of hospitalized older adult patients with medical conditions [6]. OHAT is an assessment tool designed for people with cognitive impairment and is easy to use by nondental staff, such as nurses and caretakers. Nonetheless, OHAT has drawbacks, including nonsignificant and weak correlations and percent agreements for the saliva, oral cleanliness, and dental pain categories [10]. The Oral Health Screening Tool for Nursing Personnel (OHSTNP) evaluates the functioning of the mouth as well as oral health status for inhabitants in long-term care homes. Although the OHSTNP had high specificity for screening the lips, tongue, gums, tissues, saliva, and oral cleanliness, its sensitivity is low, indicating the need for further improvements in these categories [20].

The Minimum Data Set (MDS) is a standardized assessment tool used extensively in long-term care settings that includes an oral health component. Nevertheless, studies have indicated that oral and dental items in the MDS 2.0 lack validity and often underdetect oral health problems among residents of nursing homes [21]. This limitation highlights the importance of developing an instrument like BOHAT, which can both assess oral health conditions with relative accuracy and, most importantly, provide actionable findings for nondental health care workers. The Dental Hygiene Registration (DHR) is a dental hygiene assessment scale suitable for nurses working in institutions [22]. The DHR index requires individual judgment and attendance by a dental professional. The DHR is not an alternative to examinations by dental professionals but an aid for nurses and other caregivers in their daily work [22]. The Oral Assessment Sheet aims to improve the oral health of older adults who require nursing care using 3 items in each of the following 3 categories: oral hygiene, biting and chewing, and oral function [23]. However, challenges concerning its use for oral assessments by care workers have been highlighted [23]. BOHAT seeks to fill these gaps by providing a more credible and valid assessment framework

Within nondental health care settings in India, a critical issue has emerged surrounding the insufficient attention given to oral health due to a lack of adequate training, lack of tools, and underutilization of health care providers for oral care [24]. The role of AYUSH practitioners as nondental health professionals is often underappreciated or even underutilized in the Indian health care setting. Josyula et al [25] pointed out the discrepancies in role perceptions among various health system actors, indicating that AYUSH practitioners are not fully integrated into the public health framework, which limits their ability to effectively contribute to oral health promotion. This lack of integration is echoed in the findings of Kharbanda et al [18], who emphasized that AYUSH professionals, when appropriately empowered with training, can go a long way in promoting oral health. CHWs have a role in addressing oral health issues, but their effectiveness is hindered by poor training and inadequate resources.

According to Najmunnisa et al [26], CHWs can be empowered to serve as oral health literacy workers, but actual knowledge and attitudes toward oral health are often insufficient for promoting preventive behaviors. Reddy and Singh [27] also noted that a comprehensive spectrum of health-promoting behaviors, including oral health, is central to community well-being. The necessity of integrating oral health education in the training of CHWs has thus been demonstrated by these studies, which have shown positive results after CHWs had appropriate structured training [28]. Promotion of oral health by professionals not trained as dentists and by nurses and midwives has also been documented. Villarosa et al [29] discussed how indigenous health workers promote oral health while a woman is pregnant, and their findings suggest that nondental professionals could positively impact maternal oral health. Likewise, only one study conducted by Garry and Boran [30] considered that, to improve the care of older adult patients, enhanced oral health training needs to be incorporated within the nursing curriculum. With a more concrete educational foundation, oral health outcomes could be improved across a variety of demographics.

The challenges that nondental health care providers face in India are not unique to that country. International studies conducted by McGrath et al [31] and Scrine et al [32] showed that health professionals from all disciplines acknowledge their role in oral health, yet they lack the training and resources to effectively develop programs. Therefore, there is a universal demand for cross-disciplinary collaboration and training to ensure that oral health receives adequate emphasis in broader health initiatives.

Insufficient research has been conducted to determine how oral health assessment tools can be integrated into the routine practice of nondental health care professionals. Furthermore, the existing literature does not adequately address the training needs to attain effective tool use among these professionals. Evidence pertaining to the validation of the oral health assessment tools in varying clinical scenarios and patient populations is also lacking. Finally, there is a lack of literature investigating the effectiveness of enhanced oral health assessments on overall patient outcomes and preventive care. These gaps in research provide the rationale for the development and validation of BOHAT to fill these gaps with a culturally appropriate tool that can be efficiently used by nondental health care professionals in India.

Beyond dentistry, oral health is important for general well-being and good health [33]. Unfortunately, the absence of specialized instrumentation for nondental health care providers often creates a barrier to early detection and treatment of oral health diseases. This calls for a comprehensive and easy-to-use instrument; hence, the need exists for BOHAT. It allows nondental health care professionals to conduct effective oral health assessments, thus addressing the need for holistic approaches to health care.

### Challenges and Solutions

A major challenge can be a lack of awareness of the importance of oral health care among nondental health professionals. There is a need to conduct an awareness drive with campaigns aimed at professional groups and the community. Conducting a media campaign on television, radio, print media, and social media regarding the importance of oral health and its interlinkage with general health will be very useful. Another challenge is resources and funding. A possible solution for this challenge can be the exploration of grants aimed at health care innovations and public health improvements. Integration into current health care systems presents yet another possible obstacle. Integration issues can be identified and dealt with by working closely with health care administrators to ensure the integration of BOHAT into existing workflows, developing clear protocols and guidelines on implementation, then piloting the program in a few centers prior to a wider area. There can be difficulties relating to the collection and management of data. These may be overcome by ensuring strong systems for data management and error-free and effective data collection, training health professionals in data entry and management, and digitizing tools for data collection and analyses. For its sustainability, this program will require a long-term sustainability plan that includes periodic training and follow-up support for health professionals. A monitoring and evaluation framework should also be developed to check the program’s impact, make necessary adjustments, and create community ownership and involvement to help sustain interest and participation.

### Strengths of the Study

The BOHAT study fosters holistic health care that is interdisciplinary in nature by its inclusion of nondental health care professionals, therefore providing the basis for oral health to become part of general health assessments. It builds the capacity of nondental health professionals with training and education for better health outputs. This study aims to enhance the capacity of nondental health professionals in relation to the identification and referral of oral health conditions and increase access to dental care, particularly in underserved areas. This way, early identification and intervention will help prevent the progression of these oral diseases and reduce the health care system burden—improving the quality of life of patients. The research conducted here contributes to many sustainable development goals (SDGs) that pertain to good health, quality education, reduced inequalities, and effective partnerships.

### Link to SDGs

Oral health assessments are crucial for identifying problems early and preventing more severe oral and general health conditions [34]. Therefore, BOHAT is linked with many SDGs. BOHAT will support the attainment of several health and well-being SDGs. It will help support SDG 3: “Good health and well-being” by providing a systematic approach for performing oral health assessments [35]. It will help with the early diagnosis and prevention of oral diseases by building the capacity of nondental health professionals, hence promoting health and well-being. This study aligns with SDG 4: “Quality education” by training nondental health care professionals on the use of BOHAT and promoting continued education and capacity building, ensuring that health care professionals have the proper skills to manage oral health [36]. The study will help reduce inequalities in oral health, working in concert with SDG 10: “Reduced inequalities” [37]. Hence, it will reduce health disparities in oral health outcomes but ensure equitable access to quality health care. This will add depth to the exploration of oral health practices within a specified region, like Mangaluru Taluk, and establish ways to reduce health inequalities. Thus, working with the main stakeholders—nondental health professionals, PHCs, and people within the community—shall further stress the importance of partnership in line with SDG 17: “Partnership for goals” [38]. This will help underscore the indispensability of collaborative efforts in securing SDGs.

### Research Timeline

**Figure 3:**
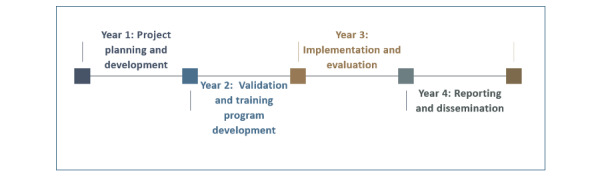
Research timeline.

Figure 3 shows the research timeline for the proposed study including the order of the main activities and the study milestones over time. The developed timeline spans a duration of 42 months, making sure that each of the stages of implementing such a research project takes place in a highly systematic, organized, and timely manner, making it easy to adapt to changes and solve eventual problems.

### Conclusion

The BOHAT study holds considerable potential for promoting oral health care through collaborative and interdisciplinary approaches. It will facilitate early diagnosis, timely referrals, and comprehensive care by integrating assessment actions for oral health into routine practice by nondental primary health care professionals. This work is a large step toward the integration of oral health into general health care, by emphasizing general health and well-being and the reduction of health disparities. This study can, therefore, serve as a model for similar efforts elsewhere in the world and help to ensure good oral health outcomes and excellent quality of life for diverse populations.

## Abbreviations

AUC: area under the curve
AYUSH: Ayurveda, yoga, naturopathy, Unani, Siddha, and homeopathy
BOHAT: Basic Oral Health Assessment Tool
CHW: community health worker
COSMIN: Consensus-Based Standards for the Selection of Health Measurement Instruments
CVI: content validity index
DHR: Dental Hygiene Registration
KAP: knowledge, attitude, and practice
MDS: Minimum Data Set
OHAT: Oral Health Assessment Tool
OHSTNP: Oral Health Screening Tool for Nursing Personnel
PHC: Primary Health Center
SDG: sustainable development goal
THROAT: the Holistic Reliable Oral Assessment Tool
WHO: World Health Organization
